# Crystal structure of flucetosulfuron

**DOI:** 10.1107/S2056989017012737

**Published:** 2017-09-12

**Authors:** Hyunjin Park, Jineun Kim, Eunjin Kwon, Tae Ho Kim

**Affiliations:** a Department of Chemistry (BK21 plus) and Research Institute of Natural Sciences, Gyeongsang National University, Jinju 52828, Republic of Korea

**Keywords:** crystal structure, herbicides, pyrimidinyl­sulfonyl­urea herbicide, flucetosulfuron

## Abstract

The title compound, {systematic name: 1-[3-({[(4,6-di­meth­oxy­pyrimidin-2-yl)carbamo­yl]amino}­sulfon­yl)pyridin-2-yl]-2-fluoro­propyl 2-meth­oxy­acetate}, C_18_H_22_FN_5_O_8_S, is used as a herbicide (pyrimidinyl­sulfonyl­urea herbicide). The dihedral angle between the mean planes of the pyridine and pyrimidine rings is 86.90 (7)°. In the crystal, N/C—H⋯O hydrogen bonds, C—H⋯F and C—H⋯π inter­actions link adjacent mol­ecules, forming a chain along [020]. A further two C—H⋯O hydrogen bonds together with weak π–π inter­actions [ring centroid separation = 3.7584 (12) Å] further aggregate the structure into a three-dimensional architecture.

## Abstract 2

The title compound, C_18_H_22_FN_5_O_8_S, is used as a herbicide. The crystal structure is stabilized by N/C—H⋯O hydrogen bond, C—H⋯F and C—H⋯π inter­actions with weak π–π inter­actions contacts to form a three-dimensional architecture.

## Chemical context   

Flucetosulfuron, a relatively new herbicide, inhibits acetolactate synthase (ALS) in plants, as do other ALS inhibitors such as imidazolinones, pyrimidinyloxybenzoates, triazolo­pyrimidines, and sulfonyl­amino­carbonyl­triazolinones (Lee *et al.*, 2014 ▸). It is a novel post-emergence sulfonyl­urea herbicide providing excellent control of *Galium aparine* and other important broadleaf weeds with good safety to cereal crops, wheat and barley (Kim, Lee *et al.*, 2003 ▸) In rice, the herbicide provides excellent control of *Echinochloa crus-galli*, which is not or only marginally controlled by common sulfonyl­urea products, and also controls annual broadleaf weeds, sedges and perennial weeds of rice with similar efficacy to other sulfonyl­urea rice herbicides (Kim, Koo *et al.*, 2003 ▸). Until now, its crystal structure had not been reported and we describe it herein.
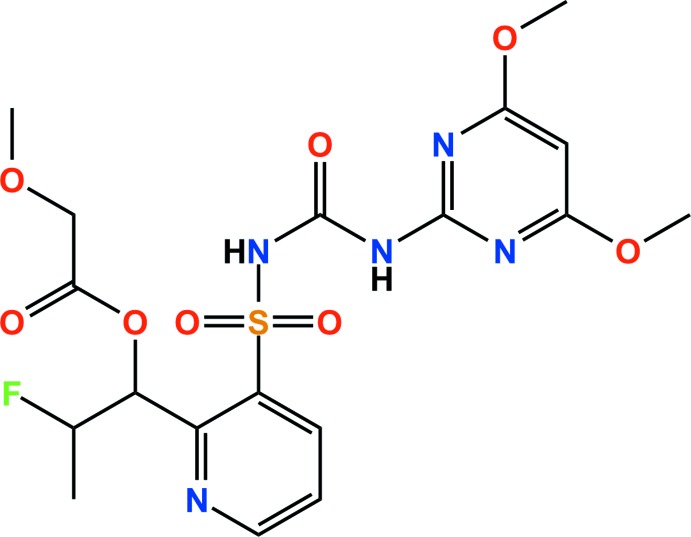


## Structural commentary   

**Figure 1:**
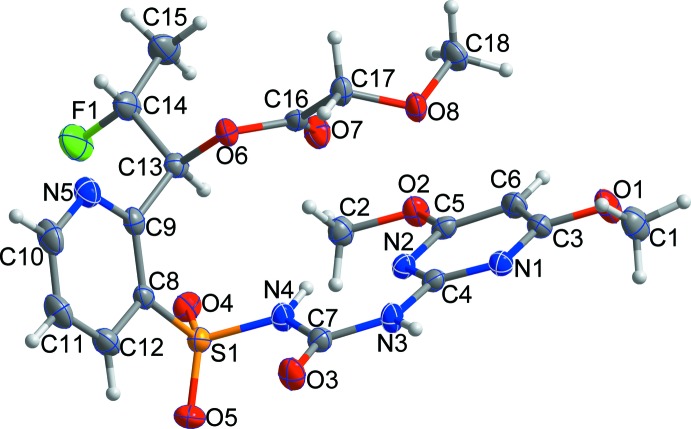
The mol­ecular structure of the title compound with the atom labelling and displacement ellipsoids drawn at the 50% probability level. H atoms are shown as small spheres of arbitrary radius.

The structure of flucetosulfuron is shown in Fig. 1 ▸. The dihedral angle between the mean planes of the pyridine and pyrimidine rings is 86.90 (7)°. All bond lengths and angles are normal and comparable to those observed in similar crystal structures (Jeon *et al.*, 2015 ▸; Chopra *et al.*, 2004 ▸).

## Supra­molecular features   

**Figure 2:**
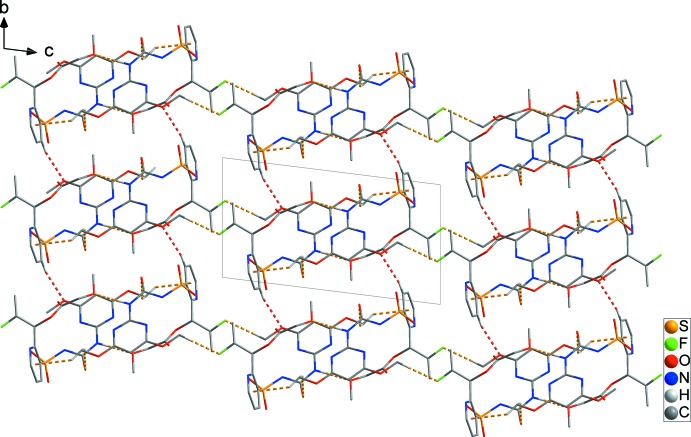
The N/C—H⋯O hydrogen bond, C—H⋯F and C—H⋯π inter­actions (yellow dashed lines) link adjacent mol­ecules, forming chains along [020]. The chains are further linked by C—H⋯O hydrogen bonds (red dashed lines), forming a two-dimensional network parallel to (020). H atoms have been omitted for clarity.

**Figure 3:**
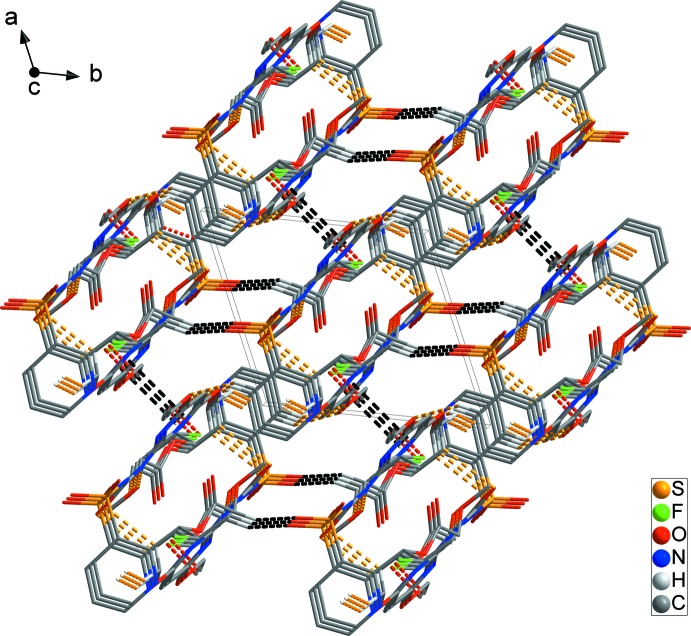
A packing diagram showing the three-dimensional architecture formed by inter­molecular C—H⋯O hydrogen bonds (red dashed lines) and π–π inter­actions (black dashed lines). H atoms have been omitted for clarity.

In the crystal, mol­ecules are linked by C1—H1*A*⋯O3^i^, N3—H3*N*⋯O8^i^ and C2—H2*B*⋯F1^ii^ hydrogen bonds [H⋯O = 2.58, 2.01 and H⋯F = 2.53 Å; Table 1 ▸] and C1—H1*B*⋯*Cg*1^i^ inter­actions [H⋯π = 2.74 Å], forming a chain structure along [020] (yellow dashed lines in Fig. 2 ▸). In addition, the chains are linked by C12—H12⋯O2^iii^ hydrogen bonds [H⋯O =2.42 Å], forming a two-dimensional network structure parallel to (020) (red dashed lines in Fig. 2 ▸). The C17—H17⋯O5^iv^ hydrogen bond [H⋯O =2.55 Å] and weak π–π inter­actions (N1–N2/C3–C6) [*Cg*2⋯*Cg*2^v^= 3.7584 (12) Å; symmetry code: (v) −*x* + 2, −*y* + 1, −*z* + 1] generate a three-dimensional architecture with mol­ecules stacked along the *a*-axis direction (black dashed lines in Fig. 3 ▸).

## Database survey   

We have reported the crystal structures of several pesticides including compounds with pyrimidinyl­sulfonyl­urea, di­meth­oxy­pyrimidin and sulfonyl­urea ring (Kang *et al.*, 2015 ▸; Jeon *et al.*, 2015 ▸; Kwon *et al.*, 2016 ▸). Moreover, a database search (CSD Version 5.27, last update February 2017; Groom *et al.*, 2016 ▸) yielded other comparable structures, methyl 2-{[3-(4,6-di­meth­oxy­pyrimidin-2-yl)ureido]sulfonyl­meth­yl}benzoate (Xia *et al.*, 2008 ▸), 2-amino-4,6-di­meth­oxy­pyrimidin-1-ium 2,2-di­chloro­acetate (Lin *et al.*, 2012 ▸), *N*-[(perhydro­cyclo­penta­[*c*]pyrrol-2-yl)amino­carbon­yl]-*o*-toluene­sulfonamide (Wu *et al.*, 2012 ▸) and 4-{4-[*N*-(5,6-di­meth­oxy­pyrimidin-4-yl)sulfamo­yl]phenyl­carbamo­yl}-2,6-di­meth­oxy­phenyl acetate (Pan *et al.*, 2012 ▸).

## Synthesis and crystallization   

The title compound was purchased from Dr Ehrenstorfer GmbH. Colourless single crystals suitable for X-ray diffraction were obtained from a CH_3_CN solution by slow evaporation at room temperature.

## Refinement   

Crystal data, data collection and structure refinement details are summarized in Table 2 ▸. All H atoms were positioned geometrically and refined using a riding model with *d*(N—H) = 0.88 Å, *U*_iso_ = 1.2*U*_eq_(C) for urea N—H, *d*(C—H) = 0.95 Å, *U*_iso_ = 1.2*U*_eq_(C) for aromatic C—H, *d*(C—H) = 0.98 Å, *U*_iso_ = 1.5*U*_eq_(C) for methyl groups, *d*(C—H) = 0.99 Å, *U*_iso_ = 1.2*U*_eq_(C) for CH_2_ group, *d*(C—H) = 1.00 Å, *U*_iso_ = 1.5*U*_eq_(C) for C*sp* ^3^—H.

## Supplementary Material

Crystal structure: contains datablock(s) I, New_Global_Publ_Block. DOI: 10.1107/S2056989017012737/hg5495sup1.cif

Structure factors: contains datablock(s) I. DOI: 10.1107/S2056989017012737/hg5495Isup2.hkl

CCDC reference: 1572854

Additional supporting information:  crystallographic information; 3D view; checkCIF report

## Figures and Tables

**Table 1:** Hydrogen-bond geometry (Å, °) *Cg*1 is the centroid of the N5/C8–C12 ring.

*D*—H⋯*A*	*D*—H	H⋯*A*	*D*⋯*A*	*D*—H⋯*A*
N3—H3*N*⋯O8^i^	0.88	2.01	2.885 (2)	174
C1—H1*A*⋯O3^i^	0.98	2.58	3.368 (3)	137
C2—H2*B*⋯F1^ii^	0.98	2.53	3.161 (2)	122
C12—H12⋯O2^iii^	0.95	2.42	3.229 (3)	143
C17—H17*A*⋯O5^iv^	0.99	2.55	3.367 (3)	139
C1—H1*B*⋯*Cg*1^i^	0.98	2.74	3.488 (2)	134

Symmetry codes: (i) 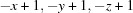; (ii) 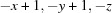; (iii) 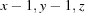; (iv) 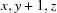.

**Table 2:** Experimental details

Crystal data	
Chemical formula	C_18_H_22_FN_5_O_8_S
*M*_r_	487.46
Crystal system, space group	Triclinic, *P* 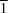
Temperature (K)	173
*a*, *b*, *c* (Å)	8.3993 (3), 9.1030 (3), 15.6862 (5)
α, β, γ (°)	92.116 (2), 101.113 (2), 112.810 (2)
*V* (Å^3^)	1076.53 (6)
*Z*	2
Radiation type	Mo *K*α
μ (mm^−1^)	0.22
Crystal size (mm)	0.36 × 0.06 × 0.05

Data collection	
Diffractometer	Bruker APEXII CCD
Absorption correction	Multi-scan (*SADABS*; Bruker, 2014 ▸)
*T*_min_, *T*_max_	0.702, 0.746
No. of measured, independent and observed [*I* > 2σ(*I*)] reflections	10919, 3773, 3081
*R*_int_	0.031
(sin θ/λ)_max_ (Å^−1^)	0.595

Refinement	
*R*[*F* ^2^ > 2σ(*F* ^2^)], *wR*(*F* ^2^), *S*	0.039, 0.099, 1.06
No. of reflections	3773
No. of parameters	302
H-atom treatment	H-atom parameters constrained
Δρ_max_, Δρ_min_ (e Å^−3^)	0.45, −0.39

Computer programs: *APEX2* and *SAINT* (Bruker, 2014 ▸), *SHELXS97* and *SHELXTL* (Sheldrick, 2008 ▸), *SHELXL2014* (Sheldrick, 2015 ▸), *DIAMOND* (Brandenburg, 2010 ▸) and *publCIF* (Westrip, 2010 ▸).

## supplementary crystallographic information

### Crystal data

**Table:**

C_18_H_22_FN_5_O_8_S	*Z* = 2
*M**_r_* = 487.46	*F*(000) = 508
Triclinic, *P*1	*D*_x_ = 1.504 Mg m^−^^3^
*a* = 8.3993 (3) Å	Mo *K*α radiation, λ = 0.71073 Å
*b* = 9.1030 (3) Å	Cell parameters from 3325 reflections
*c* = 15.6862 (5) Å	θ = 2.5–26.9°
α = 92.116 (2)°	µ = 0.22 mm^−^^1^
β = 101.113 (2)°	*T* = 173 K
γ = 112.810 (2)°	Needle, colourless
*V* = 1076.53 (6) Å^3^	0.36 × 0.06 × 0.05 mm

### Data collection

**Table:**

Bruker APEXII CCD diffractometer	3081 reflections with *I* > 2σ(*I*)
φ and ω scans	*R*_int_ = 0.031
Absorption correction: multi-scan (SADABS; Bruker, 2014)	θ_max_ = 25.0°, θ_min_ = 1.3°
*T*_min_ = 0.702, *T*_max_ = 0.746	*h* = −9→9
10919 measured reflections	*k* = −10→10
3773 independent reflections	*l* = −18→18

### Refinement

**Table:**

Refinement on *F*^2^	0 restraints
Least-squares matrix: full	Hydrogen site location: inferred from neighbouring sites
*R*[*F*^2^ > 2σ(*F*^2^)] = 0.039	H-atom parameters constrained
*wR*(*F*^2^) = 0.099	*w* = 1/[σ^2^(*F*_o_^2^) + (0.0439*P*)^2^ + 0.337*P*] where *P* = (*F*_o_^2^ + 2*F*_c_^2^)/3
*S* = 1.06	(Δ/σ)_max_ < 0.001
3773 reflections	Δρ_max_ = 0.45 e Å^−^^3^
302 parameters	Δρ_min_ = −0.39 e Å^−^^3^

### Special details

**Table:**

Geometry. All esds (except the esd in the dihedral angle between two l.s. planes) are estimated using the full covariance matrix. The cell esds are taken into account individually in the estimation of esds in distances, angles and torsion angles; correlations between esds in cell parameters are only used when they are defined by crystal symmetry. An approximate (isotropic) treatment of cell esds is used for estimating esds involving l.s. planes.

### Fractional atomic coordinates and isotropic or equivalent isotropic displacement parameters (Å^2^)

**Table:**

	*x*	*y*	*z*	*U*_iso_*/*U*_eq_	
S1	0.39351 (7)	0.10312 (6)	0.18674 (3)	0.02316 (15)	
F1	0.2117 (2)	0.35690 (17)	−0.01327 (8)	0.0529 (4)	
O1	1.0327 (2)	0.78934 (17)	0.56837 (9)	0.0345 (4)	
O2	0.97933 (18)	0.63951 (17)	0.27393 (9)	0.0268 (3)	
O3	0.34138 (19)	0.08587 (18)	0.37121 (9)	0.0309 (4)	
O4	0.48165 (18)	0.18107 (17)	0.12133 (9)	0.0278 (4)	
O5	0.3822 (2)	−0.05369 (17)	0.20150 (10)	0.0330 (4)	
O6	0.26027 (17)	0.48834 (16)	0.21292 (8)	0.0226 (3)	
O7	0.55822 (19)	0.62252 (18)	0.24672 (9)	0.0302 (4)	
O8	0.53183 (18)	0.72872 (16)	0.41130 (9)	0.0262 (4)	
N1	0.8060 (2)	0.5524 (2)	0.49899 (11)	0.0227 (4)	
N2	0.7821 (2)	0.4766 (2)	0.34862 (10)	0.0213 (4)	
N3	0.5794 (2)	0.3194 (2)	0.42532 (11)	0.0249 (4)	
H3N	0.5538	0.3064	0.4771	0.030*	
N4	0.4990 (2)	0.2289 (2)	0.27617 (10)	0.0252 (4)	
H4N	0.5839	0.3209	0.2718	0.030*	
N5	−0.0364 (2)	0.2072 (2)	0.11793 (12)	0.0310 (4)	
C1	0.9641 (3)	0.7561 (3)	0.64590 (14)	0.0356 (6)	
H1A	0.8389	0.7393	0.6326	0.053*	
H1B	1.0313	0.8472	0.6918	0.053*	
H1C	0.9752	0.6593	0.6662	0.053*	
C2	0.8773 (3)	0.5257 (3)	0.19662 (13)	0.0285 (5)	
H2A	0.8717	0.4188	0.2079	0.043*	
H2B	0.9342	0.5588	0.1475	0.043*	
H2C	0.7571	0.5224	0.1821	0.043*	
C3	0.9464 (3)	0.6843 (2)	0.49563 (13)	0.0238 (5)	
C4	0.7292 (3)	0.4557 (2)	0.42344 (13)	0.0207 (4)	
C5	0.9225 (3)	0.6125 (2)	0.34821 (13)	0.0215 (5)	
C6	1.0123 (3)	0.7244 (2)	0.42113 (13)	0.0253 (5)	
H6	1.1113	0.8211	0.4205	0.030*	
C7	0.4640 (3)	0.2007 (2)	0.35825 (13)	0.0234 (5)	
C8	0.1749 (3)	0.0953 (2)	0.16496 (12)	0.0209 (5)	
C9	0.1326 (3)	0.2238 (2)	0.13956 (13)	0.0229 (5)	
C10	−0.1640 (3)	0.0645 (3)	0.12140 (15)	0.0358 (6)	
H10	−0.2836	0.0525	0.1041	0.043*	
C11	−0.1331 (3)	−0.0655 (3)	0.14834 (15)	0.0356 (6)	
H11	−0.2283	−0.1633	0.1518	0.043*	
C12	0.0406 (3)	−0.0500 (3)	0.17037 (14)	0.0303 (5)	
H12	0.0674	−0.1376	0.1889	0.036*	
C13	0.2671 (3)	0.3930 (2)	0.13829 (12)	0.0219 (5)	
H13	0.3884	0.3942	0.1447	0.026*	
C14	0.2184 (3)	0.4629 (3)	0.05544 (13)	0.0320 (5)	
H14	0.0981	0.4629	0.0509	0.038*	
C15	0.3463 (3)	0.6282 (3)	0.04812 (15)	0.0416 (6)	
H15A	0.3513	0.7036	0.0959	0.062*	
H15B	0.3069	0.6609	−0.0082	0.062*	
H15C	0.4642	0.6286	0.0519	0.062*	
C16	0.4167 (3)	0.5958 (2)	0.26289 (13)	0.0210 (5)	
C17	0.3826 (3)	0.6730 (3)	0.34038 (12)	0.0240 (5)	
H17A	0.3527	0.7642	0.3228	0.029*	
H17B	0.2799	0.5939	0.3590	0.029*	
C18	0.6598 (3)	0.8857 (3)	0.40752 (15)	0.0316 (5)	
H18A	0.7041	0.8858	0.3541	0.047*	
H18B	0.7587	0.9161	0.4588	0.047*	
H18C	0.6045	0.9628	0.4071	0.047*	

### Atomic displacement parameters (Å^2^)

**Table:**

	*U*^11^	*U*^22^	*U*^33^	*U*^12^	*U*^13^	*U*^23^
S1	0.0254 (3)	0.0223 (3)	0.0209 (3)	0.0097 (2)	0.0034 (2)	0.0019 (2)
F1	0.0876 (12)	0.0416 (9)	0.0248 (7)	0.0235 (8)	0.0082 (7)	−0.0018 (6)
O1	0.0420 (10)	0.0256 (9)	0.0233 (8)	0.0007 (7)	0.0076 (7)	−0.0027 (6)
O2	0.0279 (8)	0.0261 (8)	0.0212 (8)	0.0035 (7)	0.0090 (6)	0.0038 (6)
O3	0.0267 (8)	0.0276 (9)	0.0286 (8)	0.0001 (7)	0.0071 (7)	0.0052 (7)
O4	0.0300 (8)	0.0334 (9)	0.0232 (8)	0.0144 (7)	0.0096 (6)	0.0055 (6)
O5	0.0405 (9)	0.0229 (8)	0.0371 (9)	0.0164 (7)	0.0046 (7)	0.0044 (7)
O6	0.0216 (7)	0.0217 (8)	0.0199 (7)	0.0049 (6)	0.0032 (6)	−0.0016 (6)
O7	0.0216 (8)	0.0325 (9)	0.0329 (9)	0.0077 (7)	0.0056 (7)	−0.0022 (7)
O8	0.0252 (8)	0.0225 (8)	0.0203 (7)	0.0007 (6)	0.0004 (6)	0.0015 (6)
N1	0.0227 (9)	0.0216 (9)	0.0217 (9)	0.0069 (8)	0.0043 (7)	0.0032 (7)
N2	0.0211 (9)	0.0224 (9)	0.0186 (9)	0.0070 (8)	0.0040 (7)	0.0049 (7)
N3	0.0247 (9)	0.0261 (10)	0.0169 (9)	0.0028 (8)	0.0044 (7)	0.0040 (7)
N4	0.0259 (10)	0.0240 (10)	0.0191 (9)	0.0034 (8)	0.0038 (7)	0.0039 (7)
N5	0.0226 (10)	0.0291 (11)	0.0374 (11)	0.0090 (9)	0.0027 (8)	−0.0029 (8)
C1	0.0451 (14)	0.0327 (13)	0.0241 (12)	0.0103 (11)	0.0094 (10)	−0.0029 (10)
C2	0.0318 (12)	0.0303 (12)	0.0216 (11)	0.0106 (10)	0.0060 (9)	0.0018 (9)
C3	0.0255 (11)	0.0194 (11)	0.0240 (11)	0.0079 (9)	0.0028 (9)	0.0022 (9)
C4	0.0201 (10)	0.0211 (11)	0.0212 (11)	0.0088 (9)	0.0034 (8)	0.0062 (9)
C5	0.0217 (11)	0.0226 (11)	0.0218 (11)	0.0098 (9)	0.0061 (8)	0.0072 (9)
C6	0.0242 (11)	0.0212 (11)	0.0256 (11)	0.0042 (9)	0.0046 (9)	0.0047 (9)
C7	0.0228 (11)	0.0234 (12)	0.0231 (11)	0.0088 (10)	0.0039 (9)	0.0045 (9)
C8	0.0233 (11)	0.0186 (11)	0.0166 (10)	0.0054 (9)	0.0018 (8)	−0.0006 (8)
C9	0.0230 (11)	0.0237 (11)	0.0178 (10)	0.0059 (9)	0.0030 (8)	−0.0018 (8)
C10	0.0206 (12)	0.0335 (14)	0.0439 (14)	0.0042 (11)	0.0030 (10)	−0.0089 (11)
C11	0.0285 (13)	0.0265 (13)	0.0412 (14)	0.0001 (11)	0.0088 (10)	−0.0034 (11)
C12	0.0348 (13)	0.0212 (12)	0.0303 (12)	0.0062 (10)	0.0077 (10)	0.0018 (9)
C13	0.0267 (11)	0.0214 (11)	0.0187 (10)	0.0104 (9)	0.0063 (8)	0.0004 (8)
C14	0.0467 (14)	0.0288 (13)	0.0205 (11)	0.0157 (11)	0.0065 (10)	0.0016 (9)
C15	0.0630 (18)	0.0304 (14)	0.0261 (13)	0.0128 (12)	0.0096 (12)	0.0091 (10)
C16	0.0221 (11)	0.0171 (10)	0.0213 (11)	0.0061 (9)	0.0024 (9)	0.0063 (8)
C17	0.0206 (11)	0.0246 (11)	0.0199 (11)	0.0028 (9)	0.0026 (8)	0.0019 (9)
C18	0.0281 (12)	0.0224 (12)	0.0343 (13)	0.0002 (10)	0.0058 (10)	0.0007 (10)

### Geometric parameters (Å, º)

**Table:**

S1—O5	1.4242 (15)	C1—H1C	0.9800
S1—O4	1.4295 (15)	C2—H2A	0.9800
S1—N4	1.6369 (17)	C2—H2B	0.9800
S1—C8	1.774 (2)	C2—H2C	0.9800
F1—C14	1.397 (2)	C3—C6	1.389 (3)
O1—C3	1.343 (2)	C5—C6	1.379 (3)
O1—C1	1.437 (3)	C6—H6	0.9500
O2—C5	1.338 (2)	C8—C12	1.385 (3)
O2—C2	1.448 (2)	C8—C9	1.398 (3)
O3—C7	1.207 (2)	C9—C13	1.520 (3)
O6—C16	1.359 (2)	C10—C11	1.371 (3)
O6—C13	1.455 (2)	C10—H10	0.9500
O7—C16	1.197 (2)	C11—C12	1.383 (3)
O8—C17	1.412 (2)	C11—H11	0.9500
O8—C18	1.429 (2)	C12—H12	0.9500
N1—C3	1.329 (3)	C13—C14	1.522 (3)
N1—C4	1.336 (2)	C13—H13	1.0000
N2—C4	1.328 (2)	C14—C15	1.497 (3)
N2—C5	1.339 (2)	C14—H14	1.0000
N3—C7	1.388 (3)	C15—H15A	0.9800
N3—C4	1.389 (2)	C15—H15B	0.9800
N3—H3N	0.8800	C15—H15C	0.9800
N4—C7	1.386 (3)	C16—C17	1.510 (3)
N4—H4N	0.8800	C17—H17A	0.9900
N5—C10	1.337 (3)	C17—H17B	0.9900
N5—C9	1.341 (3)	C18—H18A	0.9800
C1—H1A	0.9800	C18—H18B	0.9800
C1—H1B	0.9800	C18—H18C	0.9800
			
O5—S1—O4	119.31 (9)	C12—C8—S1	116.68 (16)
O5—S1—N4	110.36 (9)	C9—C8—S1	123.71 (15)
O4—S1—N4	103.89 (9)	N5—C9—C8	121.03 (18)
O5—S1—C8	107.60 (9)	N5—C9—C13	113.94 (18)
O4—S1—C8	109.39 (9)	C8—C9—C13	124.95 (18)
N4—S1—C8	105.49 (9)	N5—C10—C11	124.2 (2)
C3—O1—C1	117.99 (17)	N5—C10—H10	117.9
C5—O2—C2	117.95 (16)	C11—C10—H10	117.9
C16—O6—C13	117.59 (15)	C10—C11—C12	118.0 (2)
C17—O8—C18	114.20 (16)	C10—C11—H11	121.0
C3—N1—C4	114.61 (17)	C12—C11—H11	121.0
C4—N2—C5	116.32 (17)	C11—C12—C8	118.9 (2)
C7—N3—C4	130.02 (17)	C11—C12—H12	120.5
C7—N3—H3N	115.0	C8—C12—H12	120.5
C4—N3—H3N	115.0	O6—C13—C9	105.26 (15)
C7—N4—S1	124.61 (15)	O6—C13—C14	108.51 (16)
C7—N4—H4N	117.7	C9—C13—C14	111.96 (17)
S1—N4—H4N	117.7	O6—C13—H13	110.3
C10—N5—C9	118.31 (19)	C9—C13—H13	110.3
O1—C1—H1A	109.5	C14—C13—H13	110.3
O1—C1—H1B	109.5	F1—C14—C15	108.89 (18)
H1A—C1—H1B	109.5	F1—C14—C13	105.03 (17)
O1—C1—H1C	109.5	C15—C14—C13	115.37 (19)
H1A—C1—H1C	109.5	F1—C14—H14	109.1
H1B—C1—H1C	109.5	C15—C14—H14	109.1
O2—C2—H2A	109.5	C13—C14—H14	109.1
O2—C2—H2B	109.5	C14—C15—H15A	109.5
H2A—C2—H2B	109.5	C14—C15—H15B	109.5
O2—C2—H2C	109.5	H15A—C15—H15B	109.5
H2A—C2—H2C	109.5	C14—C15—H15C	109.5
H2B—C2—H2C	109.5	H15A—C15—H15C	109.5
N1—C3—O1	119.08 (18)	H15B—C15—H15C	109.5
N1—C3—C6	124.73 (19)	O7—C16—O6	124.13 (18)
O1—C3—C6	116.20 (18)	O7—C16—C17	126.38 (18)
N2—C4—N1	126.77 (18)	O6—C16—C17	109.49 (17)
N2—C4—N3	117.95 (17)	O8—C17—C16	111.44 (17)
N1—C4—N3	115.28 (17)	O8—C17—H17A	109.3
O2—C5—N2	118.40 (17)	C16—C17—H17A	109.3
O2—C5—C6	118.68 (18)	O8—C17—H17B	109.3
N2—C5—C6	122.91 (18)	C16—C17—H17B	109.3
C5—C6—C3	114.59 (19)	H17A—C17—H17B	108.0
C5—C6—H6	122.7	O8—C18—H18A	109.5
C3—C6—H6	122.7	O8—C18—H18B	109.5
O3—C7—N4	123.77 (18)	H18A—C18—H18B	109.5
O3—C7—N3	121.97 (18)	O8—C18—H18C	109.5
N4—C7—N3	114.23 (18)	H18A—C18—H18C	109.5
C12—C8—C9	119.55 (19)	H18B—C18—H18C	109.5
			
O5—S1—N4—C7	−48.10 (19)	O4—S1—C8—C9	−42.99 (19)
O4—S1—N4—C7	−177.12 (16)	N4—S1—C8—C9	68.21 (18)
C8—S1—N4—C7	67.84 (19)	C10—N5—C9—C8	0.1 (3)
C4—N1—C3—O1	179.93 (18)	C10—N5—C9—C13	−176.75 (18)
C4—N1—C3—C6	0.4 (3)	C12—C8—C9—N5	−1.9 (3)
C1—O1—C3—N1	−2.3 (3)	S1—C8—C9—N5	175.19 (15)
C1—O1—C3—C6	177.23 (18)	C12—C8—C9—C13	174.60 (19)
C5—N2—C4—N1	−3.1 (3)	S1—C8—C9—C13	−8.3 (3)
C5—N2—C4—N3	177.34 (17)	C9—N5—C10—C11	2.1 (3)
C3—N1—C4—N2	2.1 (3)	N5—C10—C11—C12	−2.4 (3)
C3—N1—C4—N3	−178.32 (17)	C10—C11—C12—C8	0.5 (3)
C7—N3—C4—N2	−4.5 (3)	C9—C8—C12—C11	1.6 (3)
C7—N3—C4—N1	175.92 (19)	S1—C8—C12—C11	−175.75 (16)
C2—O2—C5—N2	4.5 (3)	C16—O6—C13—C9	137.43 (16)
C2—O2—C5—C6	−176.37 (17)	C16—O6—C13—C14	−102.57 (19)
C4—N2—C5—O2	−179.30 (17)	N5—C9—C13—O6	70.7 (2)
C4—N2—C5—C6	1.6 (3)	C8—C9—C13—O6	−106.1 (2)
O2—C5—C6—C3	−178.55 (17)	N5—C9—C13—C14	−47.0 (2)
N2—C5—C6—C3	0.6 (3)	C8—C9—C13—C14	136.2 (2)
N1—C3—C6—C5	−1.6 (3)	O6—C13—C14—F1	−174.62 (16)
O1—C3—C6—C5	178.85 (17)	C9—C13—C14—F1	−58.9 (2)
S1—N4—C7—O3	−6.7 (3)	O6—C13—C14—C15	65.5 (2)
S1—N4—C7—N3	175.33 (14)	C9—C13—C14—C15	−178.77 (19)
C4—N3—C7—O3	177.7 (2)	C13—O6—C16—O7	4.0 (3)
C4—N3—C7—N4	−4.4 (3)	C13—O6—C16—C17	−175.72 (16)
O5—S1—C8—C12	3.21 (18)	C18—O8—C17—C16	85.3 (2)
O4—S1—C8—C12	134.21 (16)	O7—C16—C17—O8	−25.1 (3)
N4—S1—C8—C12	−114.60 (16)	O6—C16—C17—O8	154.58 (15)
O5—S1—C8—C9	−173.98 (16)		

### Hydrogen-bond geometry (Å, º)

Cg1 is the centroid of the N5/C8–C12 ring.

**Table:**

*D*—H···*A*	*D*—H	H···*A*	*D*···*A*	*D*—H···*A*
N3—H3*N*···O8^i^	0.88	2.01	2.885 (2)	174
C1—H1*A*···O3^i^	0.98	2.58	3.368 (3)	137
C2—H2*B*···F1^ii^	0.98	2.53	3.161 (2)	122
C12—H12···O2^iii^	0.95	2.42	3.229 (3)	143
C17—H17*A*···O5^iv^	0.99	2.55	3.367 (3)	139
C1—H1*B*···*Cg*1^i^	0.98	2.74	3.488 (2)	134

Symmetry codes: (i) −*x*+1, −*y*+1, −*z*+1; (ii) −*x*+1, −*y*+1, −*z*; (iii) *x*−1, *y*−1, *z*; (iv) *x*, *y*+1, *z*.
